# Fluid restrictive resuscitation with high molecular weight hyaluronan infusion in early peritonitis sepsis

**DOI:** 10.1186/s40635-023-00548-w

**Published:** 2023-09-21

**Authors:** Annelie Barrueta Tenhunen, Jaap van der Heijden, Paul Skorup, Marco Maccarana, Anders Larsson, Anders Larsson, Gaetano Perchiazzi, Jyrki Tenhunen

**Affiliations:** 1 Department of Surgical Sciences, Division of Anaesthesiology and Intensive Care, Uppsala University, Uppsala, Sweden; 2 Department of Medical Sciences, Division of Infectious Diseases, Uppsala University, Uppsala, Sweden; 3 Department of Medical Biochemistry and Microbiology, The Biomedical Center, University of Uppsala, Uppsala, Sweden; 4 Department of Medical Sciences, Division of Clinical Chemistry, Uppsala University, Uppsala, Sweden; 5 Hedenstierna Laboratory, Department of Surgical Sciences, Uppsala University, Uppsala, Sweden

**Keywords:** Animal model, Inflammation, Glycocalyx, Fluid therapy, Colloid

## Abstract

Sepsis is a condition with high morbidity and mortality. Prompt recognition and initiation of treatment is essential. Despite forming an integral part of sepsis management, fluid resuscitation may also lead to volume overload, which in turn is associated with increased mortality. The optimal fluid strategy in sepsis resuscitation is yet to be defined. Hyaluronan, an endogenous glycosaminoglycan with high affinity to water is an important constituent of the endothelial glycocalyx. We hypothesized that exogenously administered hyaluronan would counteract intravascular volume depletion and contribute to endothelial glycocalyx integrity in a fluid restrictive model of peritonitis. In a prospective, blinded model of porcine peritonitis sepsis, we randomized animals to intervention with hyaluronan (*n* = 8) or 0.9% saline (*n* = 8). The animals received an infusion of 0.1% hyaluronan 6 ml/kg/h, or the same volume of saline, during the first 2 h of peritonitis. Stroke volume variation and hemoconcentration were comparable in the two groups throughout the experiment. Cardiac output was higher in the intervention group during the infusion of hyaluronan (3.2 ± 0.5 l/min in intervention group vs 2.7 ± 0.2 l/min in the control group) (*p* = 0.039). The increase in lactate was more pronounced in the intervention group (3.2 ± 1.0 mmol/l in the intervention group and 1.7 ± 0.7 mmol/l in the control group) at the end of the experiment (*p* < 0.001). Concentrations of surrogate markers of glycocalyx damage; syndecan 1 (0.6 ± 0.2 ng/ml vs 0.5 ± 0.2 ng/ml, *p* = 0.292), heparan sulphate (1.23 ± 0.2 vs 1.4 ± 0.3 ng/ml, *p* = 0.211) and vascular adhesion protein 1 (7.0 ± 4.1 vs 8.2 ± 2.3 ng/ml, *p* = 0.492) were comparable in the two groups at the end of the experiment. In conclusion, hyaluronan did not counteract intravascular volume depletion in early peritonitis sepsis. However, this finding is hampered by the short observation period and a beneficial effect of HMW-HA in peritonitis sepsis cannot be discarded based on the results of the present study.

**Supplementary Information:**

The online version contains supplementary material available at 10.1186/s40635-023-00548-w.

## Background

Sepsis is a condition with high mortality in which a dysregulated host response to infection causes organ dysfunction [1]. While recovery depends on adequate anti-infective therapy, cardiovascular instability [2, 3] is antagonized with fluids and vasopressors/inotropes [4]. Early fluid resuscitation is crucial to reverse the deleterious effects of tissue hypo-perfusion, but excessive fluid therapy is associated with increased mortality [5, 6].

Microcirculatory perfusion disturbances are common in sepsis [7–9]. The deranged microcirculation is partly explained by endothelial dysfunction and glycocalyx degradation [9–12]. Endothelial dysfunction and glycocalyx degradation may lead to edema formation [13]. High molecular weight hyaluronan, HMW-HA (MW > 1000 kDa), is a highly hydrophilic constituent of the endothelial glycocalyx layer [14, 15] and contributes to vascular integrity [11, 16].

In states of inflammation, HMW-HA is degraded by several mechanisms [17–19] with concomitant shedding of the endothelial glycocalyx layer [20]. Exogenously administered glycosaminoglycans (HA, chondroitin sulphate) can restore shedded glycocalyx [16] and pericellular matrix [21] after hyaluronidase treatment. HA has been safely administered intravenously in humans [22] and reduces inflammation and lung injury in experimental sepsis [23].

In a previous study, we tested the effect of exogenously administered HMW-HA in peritonitis sepsis as adjuvant treatment to crystalloid fluid resuscitation. A post hoc analysis demonstrated a lower modified shock index (MSI = HR/MAP) during sepsis peritonitis in the intervention group [24]. Crystalloid infusion per se increases plasma concentration of HA [25] and recently liberal fluid resuscitation has been challenged by fluid restrictive approach. Therefore, in the present study we reduced the administered volume of crystalloid, with the hypothesis that exogenously administered HMW-HA counteracts intravascular volume depletion in sepsis and contributes to endothelial glycocalyx integrity in a fluid restrictive model.

The aim of the present study was to test if the intervention HMW-HA, without additional crystalloid resuscitation fluids, would counteract intravascular volume depletion in early peritonitis sepsis and contribute to improved blood circulation and preserved integrity of the glycocalyx.

## Materials and methods

The study was performed at the Hedenstierna Laboratory, Uppsala University, Sweden. Twenty male pigs (Sus scrofa domesticus) of mixed Swedish Hampshire and Yorkshire breeds (mean weight 30.4 ± 1.8 kg) received premedication with Zoletil Forte® (tiletamine and zolazepam) 6 mg/kg and Rompun® (xylazine) 2.2 mg/kg i.m. The animals were placed in supine position after adequate sedation was obtained. A peripheral intravenous catheter was placed in an auricular vein and a bolus of fentanyl of 5–10 µg/kg administered i.v. Anaesthesia was then maintained with ketamine 30 mg/kg/h, midazolam 0.1–0.2 mg/kg/h and fentanyl 4 µg/kg/h, in glucose 2.5% during the experiment. Rocuronium 2.5 mg/kg/h was added as muscle relaxant after adequate depth of anaesthesia was assured by absence of reaction to pain stimulus between the front hooves. Ringer’s acetate was infused i.v. at a rate of 10 ml/kg/h during the first 30 min of the protocol. The animals were under deep anaesthesia during the whole experiment (6 h of peritonitis/sepsis), including during euthanasia (100 mmol KCl i.v.). Bolus doses of 100 µg fentanyl i.v. were administered if signs of distress or reaction to pain stimulus were noted.

The airway of the animals was secured via tracheostomy. A tube of an internal diameter of eight mm (Mallinckrodt Medical, Athlone, Ireland) was inserted in the trachea. Thereafter, volume-controlled ventilation (Servo I, Maquet, Solna, Sweden) was maintained as follows: respiratory rate (RR) 25/min, tidal volume (*V*_T_) 8 ml/kg, positive end-expiratory pressure (PEEP) 8 cmH_2_O and inspired oxygen concentration (F_I_O_2_) 0.3. The settings of *V*_T_ and PEEP were maintained throughout the protocol, while RR was adjusted aiming at a PaCO_2_ < 6.5 kPa, and F_I_O_2_ was adjusted to keep PaO_2_ > 10 kPa.

A pulmonary artery catheter for measurement of pulmonary artery pressures and cardiac output (CO) and a triple lumen central venous catheter for fluid infusions were inserted via the right jugular vein. An arterial catheter was inserted via the right carotid artery for blood pressure measurement and blood sampling. A PiCCO (Pulse index continuous cardiac output) catheter (Pulsion, Munich, Germany) was inserted via the right femoral artery for estimation of stroke volume variation (SVV) and extravascular lung water (EVLW). Blood gases were analysed immediately after sampling and executed on an ABL 3 analyser (Radiometer, Copenhagen, Denmark). Hemoglobin (hgb) and hemoglobin oxygen saturation were analysed with a hemoximeter OSM 3 calibrated for porcine hemoglobin (Radiometer, Copenhagen, Denmark).

A midline laparotomy was performed. The bladder was catheterized for urinary drainage and an incision was made in the caecum, feces were collected and thereafter the incision in the cecal wall was closed. After insertion of a large-bore intra-peritoneal drain, the abdominal incision was closed.

### Study protocol

To yield a stock solution of 1% (10 mg/ml), five grams of HMW-HA 1560 kDa (Sodium hyaluronate Lot# 027362 HA15M-5, Lifecore Biomedical LCC, Chaska, MN, USA) was dissolved in 500 ml 0.9% saline. The solution of 1% HMW-HA was produced under sterile condition in laminar airflow and stored as 50 ml aliquots at − 20 °C. On the day of experiment, aliquots were diluted 1:10 in 0.9% saline, to yield 0.1% concentration.

After the laparotomy and collection of feces, baseline measurements were performed, after which peritonitis was induced via peritoneal instillation of autologous feces (2 g/kg body weight in 200 ml warmed 5% glucose solution). Thereafter the abdominal wall was closed.

### Experimental design

**Fig. 1:**
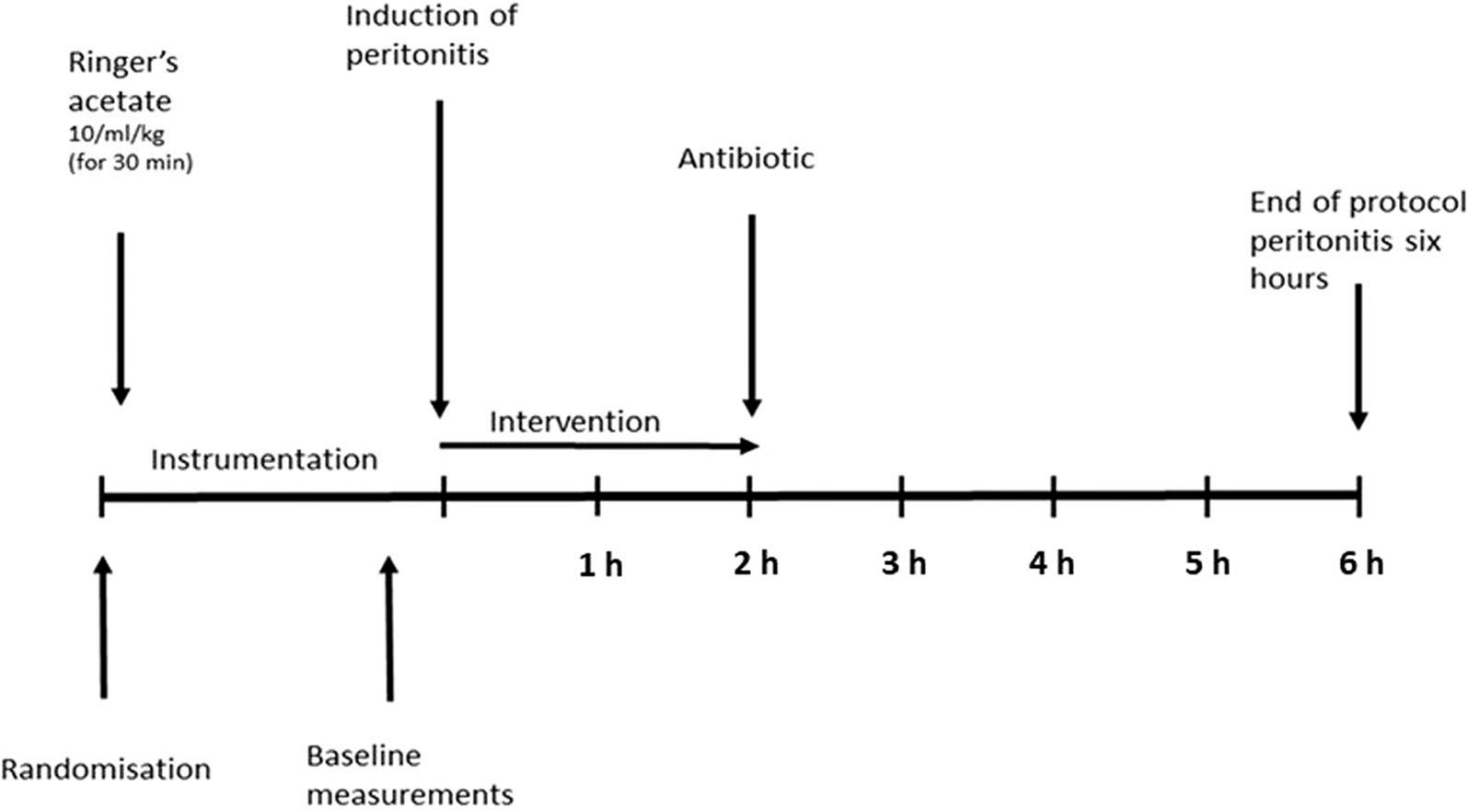
Experimental timeline. Induction of peritonitis, followed by intervention (2-h infusion) and a total of 6 h of observation period after peritonitis induction (end of protocol)

The experimental time line is presented in Fig. 1. The animals were randomized in two steps (block randomization, sealed opaque envelope), first to peritonitis (*n* = 16) or time control (*n* = 4), then into two treatment groups: intervention with HMW-HA (*n* = 8) or control group (*n* = 8). The study was prospective and the researchers were blinded for the group allocation until a master file (Additional file 2) for the whole experiment was produced.

The intervention was started at the time of the laparotomy with 0.1% solution HMW-HA, administered with a rate of 6 mg/kg/h (6 ml/kg/h) for 2 h [22]. The control group received the same volume of vehicle (0.9% saline, 6 ml/kg/h) as an infusion over 2 h. After 2 h duration of peritonitis 2 g of Piperacillin/Tazobactam in 10 ml of 0.9% saline was administered i.v.

If the animals developed circulatory instability (defined as MAP < 55 mmHg > 5 min) an infusion of Norepinephrine (40 µg/ml) was started with the rate of 5 ml/h and increased stepwise, aiming at maintaining MAP > 55 mmHg. No additional fluids were administered.

### Analyses and physiologic parameters

The primary endpoint parameter, SVV, was measured at baseline and every hour for the following 6 h duration of the experiment, simultaneously with EVLW and arterial blood gas analysis. Concomitantly, hemodynamic parameters (systemic arterial and pulmonary arterial pressures, CO, heart rate), respiratory parameters (F_I_O2, SaO_2_, E_T_CO_2_, peak pressure, plateau pressure, dynamic and static compliance) and urine output were measured. Mixed venous blood gas analysis, collection of plasma samples and arterial blood for bacterial cultures were drawn at baseline and at peritonitis duration of 1, 2, 3 and 6 h.

### Cytokine and HA analyses, VAP1, syndecan 1, heparan sulphate

Porcine-specific sandwich ELISAs were used for the determination of TNF-α and interleukin-6 (IL-6) in plasma (DY690B (TNF-α), DY686 (IL-6), R&D Systems, Minneapolis, MN, USA). The ELISAs had total coefficient of variations (CV) of approximately 6%. A commercial ELISA kit (Hyaluronan DuoSet, DY3614, R&D Systems, Minneapolis, MN, USA) was used to measure the hyaluronan concentration. Porcine specific ELISA kits were used to analyze plasma concentration of vascular adhesion protein 1 (VAP 1) (MyBioSource cat. No. MBS9364679), syndecan 1 (MyBioSource cat. No. MBS2703970) and heparan sulphate (MyBioSource cat. No. MBS265068).

### Total protein and osmolality

Total protein was analysed using a Mindray BS380 (Mindray, Shenzhen, China) with reagents from Abbott Laboratories (7D73-22, Abbott Park, IL, USA). Osmolality was measured using an OsmoPRO Multi-Sample Micro-Osmometer (I&L Biosystems, Königswinter, Germany).

### Bacterial investigations

From a sterile arterial catheter, 0.5 ml blood was drawn for quantitative blood cultures. 100 µl was cultured on three separate cysteine lactose electrolyte deficient (CLED) agar plates and cultured at 37 °C overnight. Colony forming units (CFU) were quantified with viable count technique the following day. The median of counted CFU/mL was calculated. CFU on one of three CLED plates from a timepoint was interpreted as a contamination. More than 1 CFU/mL were considered a positive blood culture.

### Statistical analysis

To determine sample size, we used data from a previous peritonitis protocol, where the stroke volume variation was used as a guide to fluid therapy [24]. The control group had a standard deviation of ± 2% at baseline. Aiming at detecting a difference of 3 per cent units of SVV between groups, a power of 0.8 and a significance level of < 0.05 justified a sample size of eight animals in each group.

The Shapiro–Wilk’s test was used to test data for normality. The two-tailed Student’s *t* test or the Mann–Whitney *U* test were used to compare the two groups, pending distribution of data. To compare the two groups throughout the experiment we used a mixed model, with the animal as random effect. The Bonferroni correction was applied.

Data are expressed as mean ± SD or median (IQR) according to distribution of data. We conducted the statistical analyses using SPSS v. 28.0.0 software (SPSS, Inc., Chicago, IL, USA). A *p* value of < 0.05 was considered statistically significant.

## Results

All animals survived to the end of the experiment. In the intervention group as well as in the control group six of eight animals presented with circulatory instability (defined as MAP < 55 mmHg > 5 min) within the time frame of the protocol.

### Hyaluronan plasma concentration

The hyaluronan concentration was comparable in the two groups with 67 ± 14 ng/ml in intervention group and 85 ± 25 ng/ml in control group (*p* = 0.103) at baseline. In the control group no statistically significant dynamics in the hyaluronan concentration was detected (one-way ANOVA, *p* = 0.580). In the intervention group the hyaluronan concentration peaked after the 2-h infusion with 158,708 ± 57,242 ng/ml and declined to 57,801 ± 32,153 ng/ml at 6 h (*p* = 0.002).

### Hemodynamics

**Fig. 2:**
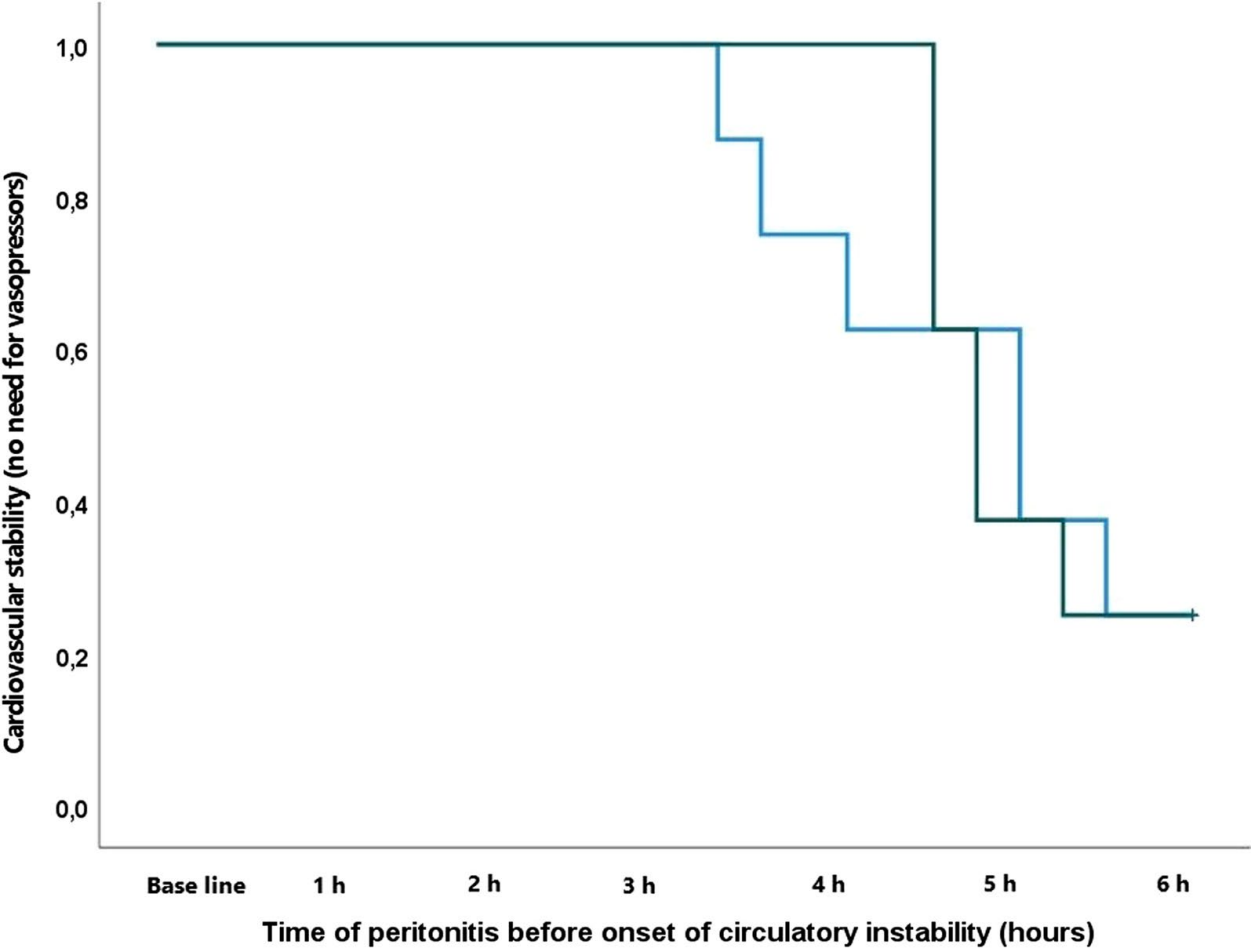
Kaplan–Meier curve depicting cardiovascular stability (no need for vasopressors) in the two groups throughout the experiment. Blue line represents intervention group and green line, control group

**Fig. 3:**
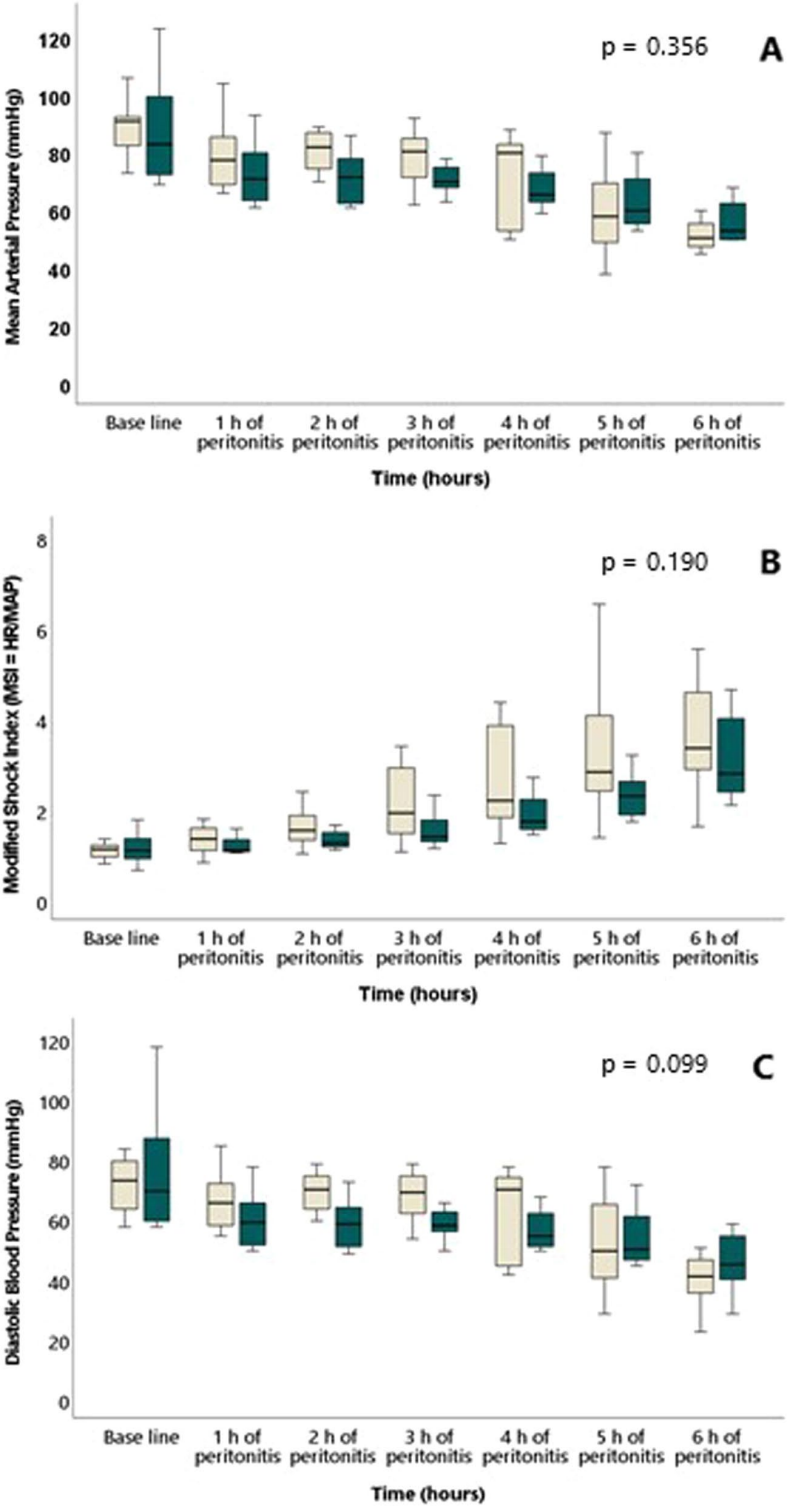
Mean arterial pressure (MAP) (**A**), Modified Shock Index (MSI = HR/MAP) (**B**) and Diastolic Blood Pressure (**C**) in the two groups throughout the experiment from baseline and hourly after induction of peritonitis. White is intervention group and green is control group. No difference between groups (mixed model)

**Fig. 4:**
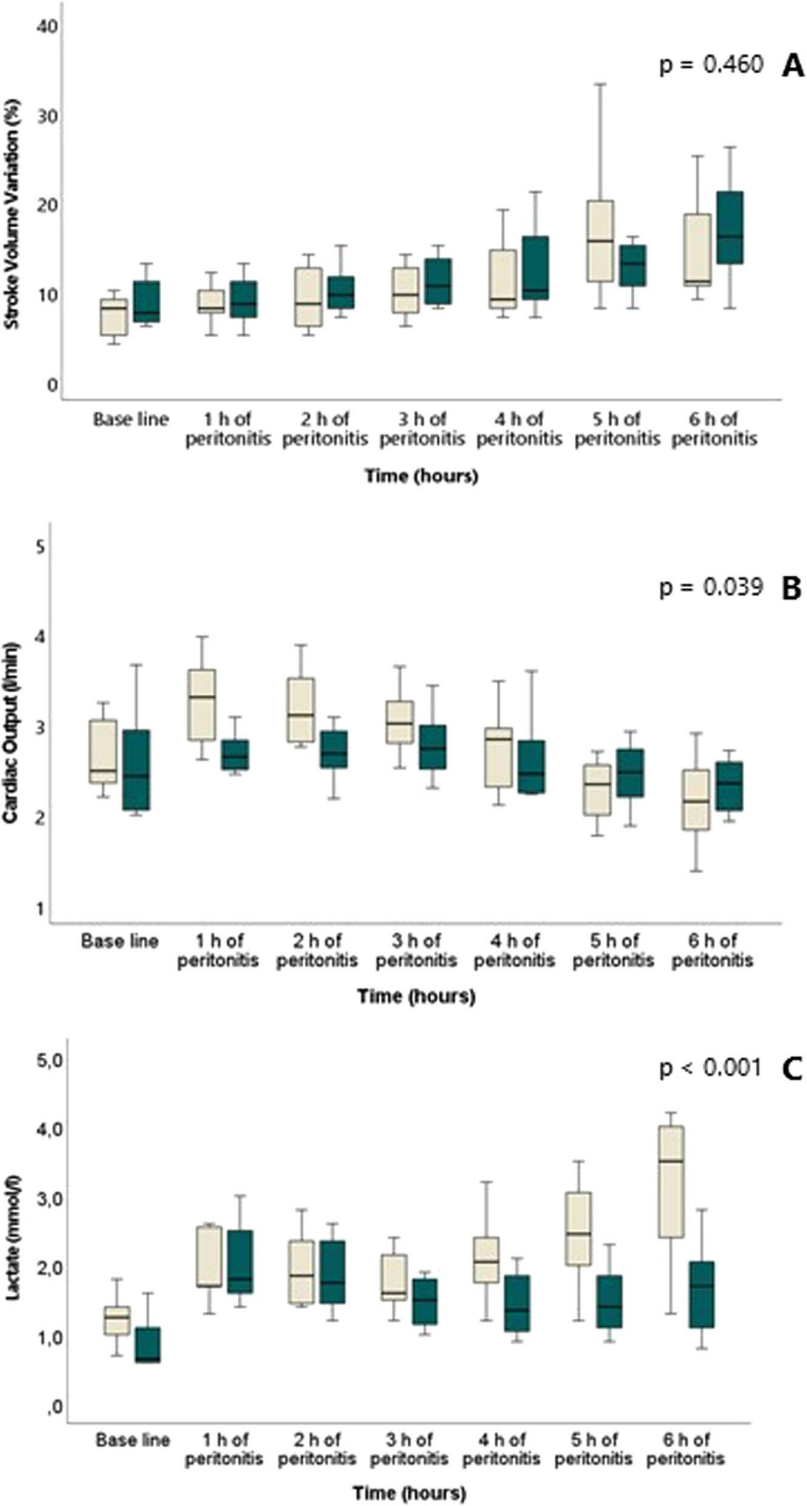
Stroke volume variation (SVV) (**A**), cardiac output (CO) (**B**) and lactate (**C**) in the two groups throughout the experiment from baseline and hourly after induction of peritonitis. White is intervention group and green is control group. No difference between groups in SVV during the experiment, CO was higher in the intervention group than in the control group during the infusion and lactate increased more in the intervention group as compared to the control group (mixed model)

Median time to onset of circulatory instability in the intervention group was 4.5 h (IQR 1.7) and 4.6 h (IQR 0.4) in the control group (*p* = 0.818) (Fig. 2). MAP declined in both groups (Fig. 3A) during the experiment, while MSI (Fig. 3B) and temperature (Table 1) increased equally in the two groups. This was associated with, and preceded by, an increase in SVV (Fig. 4A) and haemoglobin (Table 1), comparable in both groups. Diastolic blood pressure decreased comparably in the two groups as a function of time (Fig. 3C).

**Table 1:** Hemodynamics

	Group	Baseline (*n* = 8 + 8)	1 h of peritonitis (*n* = 8 + 8)	2 h of peritonitis (*n* = 8 + 8)	3 h of peritonitis (*n* = 8 + 8)	6 h of peritonitis (*n* = 8 + 8)	*p* value
BE (mEq/l)	HA	2.0 ± 3.9	2.1 ± 2.3	1.5 ± 1.7	0.5 ± 1.5	− 4.1 ± 3.3	0.061
	Control	4.6 ± 1.5	1.8 ± 2.0	1.7 ± 1.9	1.4 ± 2.0	− 1.2 ± 3.9	
CI (l/min/kg)	HA	0.09 ± 0.02	0.11 ± 0.01	0.10 ± 0.01	0.10 ± 0.01	0.07 ± 0.02	0.051
	Control	0.09 ± 0.02	0.09 ± 0.01	0.09 ± 0.01	0.09 ± 0.01	0.08 ± 0.01	
CVP (mmHg)	HA	9 ± 2	8 ± 3	8 ± 3	7 ± 3	7 ± 2	0.049^*^
	Control	9 ± 3	9 ± 3	9 ± 3	8 ± 3	7 ± 3	
ERO2	HA	0.55 ± 0.13	0.49 ± 0.09	0.50 ± 0.08	0.51 ± 0.10	0.66 ± 0.14	0.050^*^
	Control	0.48 ± 0.05	0.53 ± 0.11	0.53 ± 0.09	0.47 ± 0.09	0.60 ± 0.05	
Hb (g/l)	HA	89 ± 10	99 ± 8	110 ± 9	120 ± 10	127 ± 8	0.219
	Control	93 ± 6	100 ± 10	106 ± 13	114 ± 13	129 ± 13	
HR (BPM)	HA	94 ± 9	102 ± 21	129 ± 41	159 ± 51	178 ± 41	0.008^*^
	Control	94 ± 19	86 ± 14	96 ± 23	108 ± 26	170 ± 44	
pH	HA	7.40 ± 0.06	7.37 ± 0.04	7.35 ± 0.02	7.35 ± 0.04	7.31 ± 0.05	0.844
	Control	7.43 ± 0.02	7.37 ± 0.02	7.38 ± 0.02	7.36 ± 0.03	7.33 ± 0.07	
SV (ml)	HA	29 ± 6	33 ± 7	26 ± 8	21 ± 7	13 ± 4	0.347
	Control	28 ± 7	32 ± 3	29 ± 5	26 ± 4	14 ± 4	
SVI (ml/kg)	HA	1.0 ± 0.2	1.1 ± 0.2	0.9 ± 0.3	0.7 ± 0.2	0.4 ± 0.1	0.350
	Control	0.9 ± 0.2	1.1 ± 0.1	1.0 ± 0.1	0.9 ± 0.1	0.5 ± 0.1	
SvO2 (%)	HA	43 ± 13	48 ± 9	48 ± 8	46 ± 10	31 ± 13	0.060
	Control	50 ± 4	45 ± 11	45 ± 9	51 ± 8	37 ± 4	
T (° C)	HA	40.2 ± 0.8	40.2 ± 0.8	40.6 ± 1.0	41.1 ± 1.1	41.3 ± 1.3	0.190
	Control	39.7 ± 0.5	39.9 ± 0.8	40.2 ± 0.9	40.7 ± 1.0	41.6 ± 0.8	
Wedge Pressure (mmHg)	HA	12 ± 2	10 ± 3	10 ± 2	10 ± 2	12 ± 7	0.827
	Control	11 ± 2	11 ± 3	11 ± 3	10 ± 3	11 ± 3	

*BE* base excess, *CI* Cardiac index, *CVP* central venous pressure, *ERO*_*2*_ Oxygen extraction ratio, *Hb* hemoglobin, *HR* heart rate, *SV* stroke volume, *SVI* stroke volume index, *SvO*_*2*_ mixed venous saturation, *T* temperature. Values expressed as mean ± SD. Groups compared throughout the experiment with the mixed model analysis, *p* value of 0.05 was considered to be statistically significant, marked * in table

There was no difference in CO between intervention and control group at baseline (*p* = 0.510). CO was higher in the intervention group during the infusion of HMW-HA, but this difference between groups did not reach statistical significance when correcting for body weight (CI: l/kg/min) (Fig. 4B and Table 1). The increase in heart rate was more pronounced in the intervention group (Table 1). When comparing SV and SVI at different timepoints, there was no statistically significant difference between groups. Lactate increased in both groups from normal values at baseline, with a more pronounced increase in the intervention group during the experiment (Fig. 4C). Oxygen extraction ratio increased more in the intervention group than in the control group (Table 1) at the end of the experiment. pH and base excess (BE) were comparable in the two groups throughout the experiment. Central venous pressure (CVP) and wedge pressure were low throughout the protocol.

Norepinephrine infusion was started at onset of circulatory instability, and norepinephrine requirement was comparable in the two groups 0.42 (0.97) µg/kg/min in the intervention group vs 0.37 (0.32) µg/kg/min in control group (*p* = 0.589) as well as the weight gain of 1.8 ± 0.4 kg vs 1.8 ± 0.4 kg.

The time control animals were hemodynamically stable throughout the experiment (Additional file 1: Table S1).

### Respiratory parameters

Respiratory parameters were comparable between the two groups at baseline. Static compliance, PaO_2_/F_I_O_2_ ratio, SaO_2_, peak pressure, plateau pressure, MPAP and EVLW were comparable in the two groups throughout the experiment (Table 2).

**Table 2:** Respiratory parameters

	Group	Baseline (*n* = 8 + 8)	1 h of peritonitis (*n* = 8 + 8)	2 h of peritonitis (*n* = 8 + 8)	3 h of peritonitis (*n* = 8 + 8)	6 h of peritonitis (*n* = 8 + 8)	*p* value
Cstat (ml/cmH2O)	HA	27 ± 4	28 ± 6	25 ± 4	24 ± 4	23 ± 3	0.410
	Control	26 ± 4	24 ± 3	23 ± 3	22 ± 2	21 ± 3	
EVLW (ml)	HA	295 ± 36	316 ± 33	308 ± 33	311 ± 43	320 ± 38	0.620
	Control	308 ± 63	320 ± 59	326 ± 66	325 ± 73	358 ± 118	
MPAP (mmHg)	HA	20 ± 2	22 ± 4	23 ± 1	22 ± 2	25 ± 3	0.267
	Control	20 ± 4	18 ± 3	22 ± 3	21 ± 4	25 ± 4	
PaO2/FIO2	HA	55 ± 13	50 ± 8	49 ± 8	47 ± 8	43 ± 8	0.811
	Control	59 ± 14	52 ± 7	51 ± 6	49 ± 7	42 ± 6	
Pplat (cmH2O)	HA	18 ± 2	18 ± 2	18 ± 1	18 ± 1	19 ± 1	0.363
	Control	19 ± 2	20 ± 2	20 ± 1	19 ± 1	21 ± 1	
SaO2 (%)	HA	96 ± 1	95 ± 1	94 ± 1	94 ± 2	92 ± 2	0.813
	Control	96 ± 2	96 ± 1	96 ± 1	95 ± 1	93 ± 2	

*Cstat* static compliance, *EVLW* extra vascular lung water, *MPAP* mean pulmonary arterial pressure, *PaO2/FIO2* arterial oxygen partial pressure to fractional inspired oxygen ratio, *Pplat* plateau pressure, *SaO2* arterial oxygen saturation. Values expressed as mean ± SD. No difference between groups throughout the experiment (mixed model analysis)

### IL-6, TNFα and blood cultures

Plasma concentrations of IL-6 increased in both intervention and control groups from baseline throughout the experiment (6 h of peritonitis) from 80 (150) to 4316 (3940) pg/ml from 80 (0) to 4145 (2336) pg/ml, no difference between groups over time (*p* = 0.877). TNFα increased comparably in both groups (*p* = 0.932). Blood cultures were positive in three animals in each group during the observation period.

### Syndecan 1, heparan sulphate and VAP 1

Plasma concentration of Syndecan 1 was comparable in the two groups at 6 h of peritonitis 0.6 ± 0.2 ng/ml vs 0.5 ± 0.2 ng/ml (*p* = 0.292). Heparan sulphate concentration did not differ between the two groups 1.23 ± 0.2 vs 1.4 ± 0.3 ng/ml (*p* = 0.211).

Plasma concentration of VAP1 was at 6 h of peritonitis comparable between intervention and control groups, 7.0 ± 4.1 vs 8.2 ± 2.3 ng/ml (*p* = 0.492).

### Plasma protein and osmolality

Total protein was 40.7 (5.7) g/l in intervention group at baseline and 37.6 (3.5) g/l at 6 h of peritonitis (*p* = 0.207), vs 39.6 (5.3) g/l at baseline in the control group and 35.8 (6.0) g/l at the end of the protocol. There was no difference between groups throughout the experiment (*p* = 0.684).

Osmolality was 284 (26) mOsm/kg at baseline in intervention group and 275 (29) mOsm/kg at 6 h, vs 283 (15) mOsm/kg at baseline and 275 (8) mOsm/kg at 6 h in the control group. No difference between the groups throughout the experiment (*p* = 0.645).

## Discussion

In the present study, we opted for a fluid restrictive resuscitation strategy in a porcine model of peritonitis sepsis. We hypothesized that the intervention with HMW-HA without additional crystalloid infusion would suffice to better maintain intravascular volume, blood circulation and the integrity of the glycocalyx. We chose SVV, a dynamic surrogate marker of intravascular volume as our primary end point parameter. Contrary to our hypothesis, HMW-HA administered in the early course of porcine peritonitis did not counteract the signs of intravascular hypovolemia as depicted by increasing SVV. Hemoconcentration, increasing hgb/hematocrit (capillary leakage) and surrogate markers of endothelial glycocalyx damage (syndecan 1, heparan sulfate, VAP 1) did not differ between the two groups during the whole of the experiment. The inflammatory response as reflected by concentrations of selected cytokines in plasma was comparable in the two groups.

SVV is a validated method to assess preload responsiveness in mechanically ventilated, critically ill patients [26], with the magnitude of the cyclic changes in left ventricular stroke volume being proportional to volume responsiveness [27]. Apart from being a means of dynamic monitoring to guide fluid resuscitation, SVV can also be used to evaluate intravascular volume status [28]. In the present study no difference in SVV was observed between groups during the 6 h of peritonitis sepsis, which was in accordance with the finding of comparable increase in plasma levels of hgb between the two groups; marker of hemoconcentration and indicative of loss of intravascular volume.

Plasma concentrations of HA peaked with a mean value of 158,708 ± 57,242 ng/ml, directly after the intervention was stopped (2 h of infusion), followed by a decline already at 6 h to 57,801 ± 32,153 ng/ml. In a study by Hamilton et al. 2009 [22], the same dose (12 mg/kg as an infusion administered over 2 h) of HA (mean MW 280 kDa) in healthy volunteers resulted in a two times higher peak concentration and the decline was not as pronounced at 6 h.

Lactate increase was more pronounced in the intervention group at the end of the observation period. This increase in lactate was not accompanied by a significant difference in pH or BE between the two groups. While there are several explanations to hyperlactatemia alone in sepsis, association to metabolic acidosis is most commonly interpreted as due to cardiovascular dysfunction or tissue hypoperfusion. Hypoxic hyperlactatemia can be explained by low cardiac output states and/or volume depletion [29]. Hyperlactatemia, especially when refractory to resuscitation, is associated with increased mortality in sepsis [4, 29, 30]. In the present study, CO was higher in the intervention group during the infusion of HMW-HA. After discontinued HMW-HA infusion oxygen extraction ratio increased towards the end of the observation period, accompanied by hyperlactatemia. This may suggest that discontinuation of HMW-HA infusion may be detrimental, when no additional fluid resuscitation follows. However, in our fluid restrictive model of peritonitis, the finding of higher lactate in the intervention group is hampered by the short observation period. A more balanced resuscitation strategy might be of value to draw definite conclusions of potential benefit or harm.

Syndecan 1 and heparan sulfate in plasma are both sensitive markers of shedding of endothelial glycocalyx [31]. VAP 1 correlates with increased plasma concentrations of syndecan 1 in septic shock [32]. In our study, there was no difference in measured concentrations of syndecan 1, heparan sulfate or VAP 1 between groups. These findings suggest that HMW-HA alone does not exert a protective effect on the glycocalyx in early peritonitis sepsis. Norepinephrine requirements were also similar between the two groups as well as total protein, indicating a similar intravascular status in the two groups.

Even though our model of postoperative peritonitis with source control is clinically relevant, an animal model can never fully replicate the human sepsis syndrome, due to possible differences in host response to both insult and intervention. Another limitation of the study is the short observation period. We opted for this time frame based on previous experiments to focus on the onset of peritonitis sepsis and the initial phase of glycocalyx shedding. However, in the present study the 6 h protocol might have been too short for all the animals to develop circulatory instability. In addition, observed differences between groups, when present, were small, and not necessarily clinically relevant, even if statistically significant. Furthermore, the fluid restrictive model enabled us to minimize the potential shedding of glycocalyx from crystalloid infusion per se; however, it did not mimic the care of the septic patient, in which fluid resuscitation forms an integral part. Time control animals were stable throughout the experiment confirming the robustness of our model.

In conclusion, in this study, contrary to our hypothesis, HMW-HA infusion in a fluid restrictive model of early peritonitis sepsis did not preserve intravascular volume status. While the infusion of HMW-HA was associated with higher CO, the increase in oxygen extraction ratio accompanied by hyperlactatemia after discontinuation of the intervention casts doubts on any potential beneficial effects of HMW-HA. For future studies, a balanced resuscitation strategy should be considered, with continuous infusion of HMW-HA, possibly with the addition of chondroitin sulphate [16, 21].

### Supplementary Information

**Additional file 1: Table S1.** Time controls.

**Additional file 2.** Masterfile. Raw data.

## Data Availability

All data generated or analysed during this study are included in this article (and its Additional information files).

## Abbreviations

PaO_2_/F_I_O_2_: Arterial oxygen partial pressure/fractional inspired oxygen
BE: Base excess
CO: Cardiac output
CFU: Colony forming units
CVP: Central venous pressure
EVLW: Extravascular lung water
F_I_O_2_: Inspired oxygen concentration
HMW-HA: High molecular weight hyaluronan
HA: Hyaluronan
MAP: Mean arterial pressure
MPAP: Mean pulmonary arterial pressure
SvO_2_: Mixed venous oxygen saturation
MSI: Modified shock index
PEEP: Positive end-expiratory pressure
PiCCO: Pulse index continuous cardiac output
RR: Respiratory rate
V_T_: Tidal volume
SVV: Stroke volume variation
VAP1: Vascular adhesion protein 1
