# Supplementary material for: Fluid restrictive resuscitation with high molecular weight hyaluronan infusion in early peritonitis sepsis
Source: Intensive Care Med Exp. 2023 Sep 21;11:63. doi: 10.1186/s40635-023-00548-w (PMC10513979; doi:10.1186/s40635-023-00548-w)
Supplement: Supplementary file 1 — Additional file 1: Table S1. Time controls. [file 40635_2023_548_MOESM1_ESM.docx]

**Additional material. Table. Time controls.**

|  | **Baseline**  **(n = 2+2)** | **3 hours post**  **laparotomy**  **(n = 2+2)** | **6 hours post**  **laparotomy**  **(n = 2+2)** |
| --- | --- | --- | --- |
| MAP (mmHg) HA | 84 ± 9 | 83 ± 8 | 83 ± 20 |
| MAP(mmHg) NaCl | 95 ± 13 | 85 ± 8 | 77 ± 22 |
| MSI HA | 1.1 ± 0.3 | 1.0 ± 0.2 | 1.1 ± 0.2 |
| MSI NaCl | 1.0 ± 0.3 | 1.3 ± 0.4 | 1.3 ± 0.3 |
| HR (BPM) HA | 93 ± 18 | 80 ± 9 | 89 ± 5 |
| HR (BPM) NaCl | 100 ± 38 | 108 ± 19 | 96 ± 4 |
| CVP (mmHg) HA | 10 ± 1 | 12 ± 1 | 11 ± 3 |
| CVP (mmHg) NaCl | 10 ± 1 | 10 ± 1 | 10 ± 2 |
| CO (l/min) HA | 3.5 ± 0.8 | 2.9 ± 0.6 | 3.5 ± 0.9 |
| CO (l/min) NaCl | 3.3 ± 1.4 | 3.9 ± 0.8 | 3.3 ± 0.7 |
| EVLW (ml) HA | 304 ± 20 | 322 ± 5 | 315 ± 19 |
| EVLW (ml) NaCl | 318 ± 74 | 353 ± 45 | 349 ± 49 |
| SVV (%)HA | 9 ± 3 | 11 ± 4 | 10 ± 0 |
| SVV (%)NaCl | 10 ± 1 | 11 ± 4 | 13 ± 1 |
| MPAP (mmHg) HA | 21 ± 0 | 23 ± 2 | 23 ± 2 |
| MPAP(mmHg) NaCl | 21 ± 2 | 22 ± 1 | 21 ± 3 |
| PaO_2_/F_I_O_2_ HA | 53 ± 8 | 52 ± 9 | 49 ± 7 |
| PaO_2_/F_I_O_2_ NaCl | 54 ± 2 | 50 ± 4 | 51 ± 3 |
| Hb (g/l) HA | 93 ± 10 | 92 ± 16 | 90 ± 6 |
| Hb (g/l) NaCl | 98 ± 11 | 98 ± 4 | 101 ± 9 |
| Lactate (mmol/l) HA | 1.1 ± 0.1 | 1.3 ± 0.8 | 1.3 ± 0.6 |
| Lactate(mmol/l) NaCl | 0.9 ± 0.1 | 0.7 ± 0.3 | 0.9 ± 0.6 |
| SvO_2_ (%)HA | 53 ± 15 | 42 ± 13 | 50 ± 10 |
| SvO_2_ (%)NaCl | 44 ± 15 | 50 ± 13 | 49 ± 6 |

Mean arterial pressure (MAP), Modified shock index (MSI), Heart rate (HR), Central venous pressure (CVP), Cardiac output (CO), Extravascular lung water (EVLW), Stroke volume variation (SVV), Mean pulmonary arterial pressure (MPAP), Arterial oxygen partial pressure to fractional inspired oxygen ratio (PaO_2_/F_I_O_2_), Hemoglobin (Hb), mixed venous oxygen saturation (SvO_2_). Values reported as mean ± SD.
